# Dilation of the ascending aorta in Turner syndrome - a prospective cardiovascular magnetic resonance study

**DOI:** 10.1186/1532-429X-13-24

**Published:** 2011-04-28

**Authors:** Kristian H Mortensen, Britta E Hjerrild, Kirstine Stochholm, Niels H Andersen, Keld Ejvind Sørensen, Erik Lundorf, Arne Hørlyck, Erik M Pedersen, Jens S Christiansen, Claus H Gravholt

**Affiliations:** 1 Department of Endocrinology and Internal Medicine (MEA) and Medical Research Laboratories, Aarhus Hospital NBG, Aarhus University Hospital, Aarhus, Denmark; 2 Department of Cardiology, Aarhus University Hospital Skejby, Aarhus, Denmark; 3 The MR Centre, Aarhus University Hospital Skejby, Aarhus, Denmark; 4 Department of Radiology, Aarhus University Hospital Skejby, Aarhus, Denmark; 5 Department of Radiology, Aarhus Hospital NBG, Aarhus University Hospital, Aarhus, Denmark

## Abstract

**Background:**

The risk of aortic dissection is 100-fold increased in Turner syndrome (TS). Unfortunately, risk stratification is inadequate due to a lack of insight into the natural course of the syndrome-associated aortopathy. Therefore, this study aimed to prospectively assess aortic dimensions in TS.

**Methods:**

Eighty adult TS patients were examined twice with a mean follow-up of 2.4 ± 0.4 years, and 67 healthy age and gender-matched controls were examined once. Aortic dimensions were measured at nine predefined positions using 3D, non-contrast and free-breathing cardiovascular magnetic resonance. Transthoracic echocardiography and 24-hour ambulatory blood pressure were also performed.

**Results:**

At baseline, aortic diameters (body surface area indexed) were larger at all positions in TS. Aortic dilation was more prevalent at all positions excluding the distal transverse aortic arch. Aortic diameter increased in the aortic sinus, at the sinotubular junction and in the mid-ascending aorta with growth rates of 0.1 - 0.4 mm/year. Aortic diameters at all other positions were unchanged. The bicuspid aortic valve conferred higher aortic sinus growth rates (p < 0.05). No other predictors of aortic growth were identified.

**Conclusion:**

A general aortopathy is present in TS with enlargement of the ascending aorta, which is accelerated in the presence of a bicuspid aortic valve.

## Background

The incidence of aortic dissection is 100-fold increased in Turner syndrome (TS) [1-3], where aortic dilation, bicuspid aortic valve (BAV), aortic coarctation, karyotype 45X and hypertension confer increased risk of dissection [4-7]. However, these risk markers are not identified in all aortic dissections in TS [4,5] and aortic follow-up is therefore recommended for all patients [8].

Cross-sectional studies show a high prevalence of aortic dilation at all ages in TS [6,7,9,10] whilst knowledge of the development of the aortopathy over time is limited. Only one transthoracic echocardiography study (TTE) has prospectively assessed aortic dimensions in TS [11]. In this study the ascending aorta was seen to enlarge over 37-months but aortic growth could not be associated with the risk markers for aortic dissection in TS [11]. However, TTE is not the most likely modality to provide successful imaging in TS [12,13] where assessment of the entire aorta is of key importance because the arteriopathy extends beyond the ascending aorta [6,14] and a fifth of dissections occur in the descending aorta [4,5]. In contrast to this, 3D cardiovascular magnetic resonance (CMR) offers more optimal non-radiation imaging of the entire thoracic aorta that is applicable to most TS patients [6,13] and ideal for serial aortopathy assessment.

In the present study we set out to provide prospective data on the aortopathy in TS using highly sensitive 3D, free-breathing and contrast-free CMR [15]. By use of this modality we aimed to study aortic growth, as it is an important surrogate marker for aortic dissection that adds prognostic information to cross-sectionally assessed aortic diameter [16]. Furthermore, we aimed to elucidate associations between aortic growth and the currently used risk markers for aortic dissection.

## Methods

### Study Population

Patients with karyotypically proven TS (*n *= 102) were recruited through the Danish National Society of Turner Syndrome Contact Group and an endocrine outpatient clinic. Exclusion criteria included malignancy, liver disease and contraindications to CMR (including mechanical aortic valve prosthesis). Healthy, age-matched females (*n *= 67) were recruited by advertisement to serve as baseline controls. The patients were examined at baseline and follow-up with CMR, TTE and 24-hour ambulatory blood pressure monitoring. The controls were examined once.

The study was conducted in compliance with the Helsinki Declaration. Aarhus County Ethical Scientific Committee (Denmark) approved the trial protocol (# 20010248). All participants gave informed consented.

### Cardiovascular Magnetic Resonance Imaging

CMR was performed with a 1.5 Tesla whole-body magnetic resonance scanner (ACS-NT, Philips Medical Systems; maximum gradient performance 30 Tesla per meter amplitude, slew rate 150 Tesla/m/sec). A 5-element cardiac coil was used. After initial scouts, a 3D data stack (27 cm [AP] × 15 cm [FH] × 36 cm [LR]) covering the entire thoracic aorta was acquired. A contrast-free, nearly isotropic, fat-saturated, 3D steady-state free precession and ECG-triggered gradient echo sequence (250 ms diastolic acquisition window) with a respiratory navigator was used [6,14]. All patients were examined by the same staff and in the same scanner.

**Figure 1:**
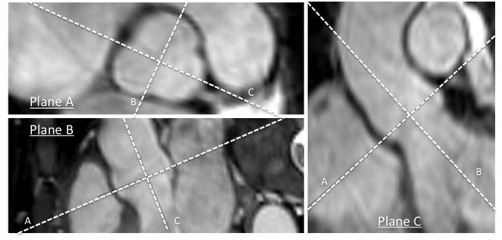
**Aortic measurements by multiplanar reformatting**. Multiplane reformatting was used to map diameters of the aorta in a measurement plane that was perpendicular to the direction of blood flow and orthogonal to the aortic wall at the position of measurement. Aortic and extra-aortic anatomical landmarks defined these positions. Here, the positioning of the measurement plane (Plane A) is schematically demonstrated for the aortic sinus in a patient with Turner syndrome and tricuspid aortic valve morphology.

Systematic analysis was performed using dedicated software (Systematic Software Engineering, Aarhus, Denmark), allowing reconstruction of the 3D stack of data in any plane (Figure 1) [6,14]. All CMR scans were reviewed for morphologic abnormalities of the aorta and the major branch arteries. Abnormalities were defined according to the previously used definitions: (i) aortic coarctation, (ii) elongated transverse aortic arch, (iii) aberrant right subclavian artery, and (iv) bovine aortic arch [14].

**Figure 2:**
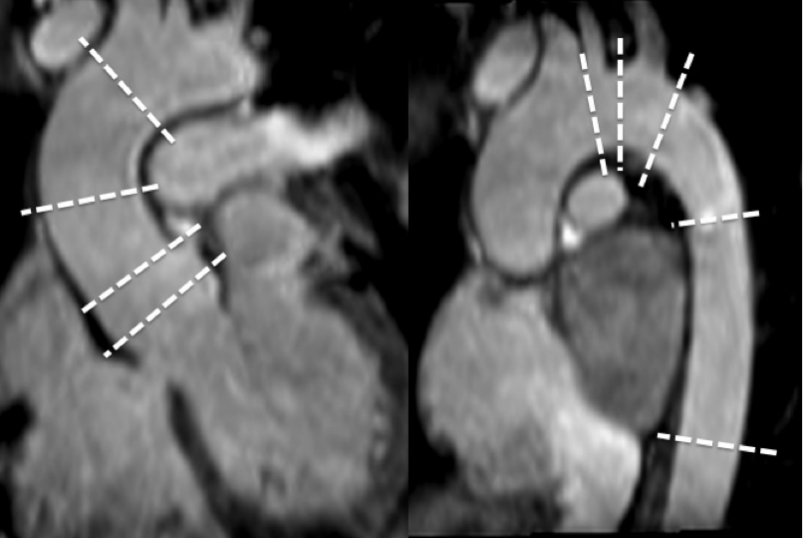
**Measurements positions from 3D CMR**. Measurements were obtained from nine positions along the thoracic aorta, spanning from aortic sinus level to the distal descending aorta. Each measurement position was adjusted using 3D multiplanar reformatting, which was guided by local aortic and extra-aortic landmarks as well as the shape and dimensions of the aortic lumen.

**Figure 3:**
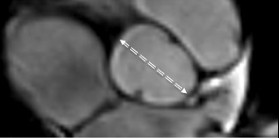
**Aortic diameter measured from 3D multiplanar reformatted CMR**. Aortic diameter was obtained as the maximum diameter of the high intensity signal of the blood pool within the vessel lumen on 3D non-contrast enhanced, balanced steady state free precession CMR. No assumptions of circular anatomy were made. At the level of the aortic sinus, cusp-to-opposing-cusp diameters were obtained for the optimum diameter assessment as shown here for the aortic sinus in a patient with Turner syndrome and bicuspid aortic valve morphology.

Two CMR experienced observers measured true crosscut, perpendicular and maximum aortic diameters (Figure 1, 2, 3). The observers were blinded to the clinical data of the patient. Aortic or extra-aortic landmarks guided the measurement positions. The positions were: (i) *aortic sinuses *(measuring cusp-to-opposing-cusp diameter at the point of the maximum aortic diameter in the aortic sinus [Figure 3]), (ii) *ascending aorta *at the sinotubular junction; (iii) *mid-ascending aorta *at the level of the inferior margin of right pulmonary artery; (iv) *distal ascending aorta *immediately proximal to brachiocephalic artery; (v) *proximal aortic arch *between the brachiocephalic and left carotid artery arteries; (vi) *distal aortic arch *immediately proximal to left subclavian artery; (vii) *aortic isthmus *immediately distal to the left subclavian artery; (viii) *proximal descending aorta *between the left pulmonary artery and the top of left atrium: and: (ix) *distal descending aorta *at the most caudal border of the left atrium. At these positions (Figure 2), two imaging planes perpendicular to each other and to the measurement plane were simultaneously displayed to ensure a correct measurement (Figure 1). All measurements were obtained as maximum intra-luminal diameter, defined by the high-intensity signal in the vessel lumen on steady state free precession CMR (Figure 3). Measurements were taken without assumptions of circular anatomy of the aortic lumen.

The baseline measurement planes (Figure 1) were stored and subsequently used to guide the re-assessment of the aortic diameters at follow-up, ensuring that follow-up measurements were taken as close as possible to the baseline measurement positions. These stored baseline images were blinded for the previously obtained measurements. Baseline and follow-up measurements were not taken at the same time from the collected data sets.

Two observers measured 20 randomly chosen CMR scans to determine interobserver measurement variability. This was carried out according to the measurement methodology described above, where one observer defined the measurement plane and the stored this plane in a blinded format (Figure 1). The second observer then re-measured the diameter using this plane to adjust their measurement position. After this, one observer re-measured these 20 scans to assess intraobserver measurement variability using the same approach. During this re-measurement the observer was blinded to the previously obtained measurements and to whether they were measuring a follow-up scan or re-measuring a baseline scan (i.e. 100 scans were assessed in random order, comprising 80 follow-up scans mixed with 20 repeat baseline measurements).

Aortic dilation was defined as a diameter exceeding two standard deviations above the mean of the control group. All measurements were indexed for body surface area (BSA) [7].

### Echocardiography and Blood pressure

One experienced observer performed TTE on a GE Vivid Seven (GE Healthcare, Horten, Norway) with a 2.5 mHz transducer using second harmonic imaging. Aortic valve morphology and function were assessed. Leaflet morphology was registered in BAV patients: fusion of right and left coronary cusp, right and non-coronary fusion, and left and non-coronary fusion [17]. Aortic stenosis was diagnosed from Doppler tracings, and classified as mild (mean gradient below 25 mm Hg), moderate (mean gradient 25 to 40 mm Hg) or severe (mean gradient above 40 mm Hg) [18]. Aortic regurgitation was defined from colour Doppler as the width of the vena contracta in the parasternal long axis view, and classified as mild (above 0.3 cm), moderate (0.3 to 0.6 cm) or severe (above 0.6 cm) [18].

Ambulatory blood pressures were recorded over 24-hours with oscillometric measurements every 20 minutes (SpaceLabs 91207, Washington, USA). The cuff was positioned on the left arm and cuff size was adjusted to the upper arm circumference.

### Statistical analyses

Statistical computations were performed using SPSS 18.0 and GraphPad Prism 5.0. Normal distribution of data was tested with Shapiro-Wilk test. Continuous variables are expressed as means ± standard deviations. Data was compared using Student's independent t-test or paired t-test. Correlations were assessed by Pearson's coefficient of correlation or binomial distribution with either the χ^2 ^test (if the expected distribution was > 5 in all cells) or Fisher's exact test (if the expected distribution was ≤ 5 in one or more cells). Backward multiple linear regression was used to examine the principal determinants of aortic growth rates, where independent variables were omitted from the model when p > 0.1. Otherwise, p < 0.05 was considered statistically significant. CMR reproducibility was tested with Bland-Altman analysis, where a linear approach was taken after ensuring that the degree of variability did not vary systematically with the measured diameters [19].

## Results

### Study population

Eighty of 102 recruited TS patients completed the follow-up. At baseline, one patient was diagnosed with chronic Stanford type A dissection, which led to exclusion from the study. Between baseline and follow-up, one patient had aortic valve replacement for severe aortic valve stenosis, and she was excluded. Three patients died during the follow-up (one from cardiogenic shock after surgery for severe aortic dilation [maximum aortic diameter 49.7 mm in the mid-ascending aorta]; one from unexplained sudden death [maximum aortic diameter 39.0 mm in the aortic sinus at baseline]; and one from an epileptic seizure). No autopsies were performed. Eight patients withdrew their consent for non-health related reasons before follow-up. No cardiovascular events occurred during follow-up in patients who completed the study or withdrew their consent. In 9 patients, either baseline or follow-up CMR scans were not usable, mainly because of poor patient compliance but in a few cases for technical reasons; all 9 patients declined repeat imaging.

The mean follow-up interval for the 80 patients with TS who completed both baseline and follow-up CMR was 2.4 ± 0.4 years (range: 1.4 - 3.5 years). Patient and control characteristics are given in Table 1, and baseline characteristics for the entire baseline cohort have been described previously [6].

**Table 1:** Demographics and descriptives in Turner syndrome and controls

	Turner syndrome		Controls
	
	n = 80		n = 67
**Baseline age **(years [range])	38 ± 10 [18 - 60]		39 ± 12 [20 - 63] ***

**Body surface area (**m^2^)	1.5 ± 0.2		1.8 ± 0.2 **

**Karyotype **(45X/non-45X, %)	60%/40%		-

**Growth hormone substitution **^**a**^			
Treated (%)	28%		-
Exposure time (years)	5 ± 3		-
**Estrogen substitution **^**a**^			
Treated (%)	85%		-
Exposure time (years)	19 ± 9		-

**Aortic abnormalities**			
Elongated transverse aortic arch	48%		-
Aortic coarctation	11%		-
Previous coarctation repair	9%		-
Aortic arch hypoplasia	2%		-
Bovine aortic arch	8%		8%
Aberrant right subclavian artery	10%		-

	**Baseline**	**Follow-up**	

**Antihypertensive treatment**	30%	46%	

**Ambulatory blood pressure**			
24-hour systolic (mm Hg)	122 ± 14	120 ± 12 *	113 ± 11 **
24-hour diastolic (mm Hg)	78 ± 11	76 ± 9 *	71 ± 8 **
24-hour heart rate (beats/min)	77 ± 9	75 ± 8 *	71 ± 9 **

Continuous variable are expressed as means ± standard deviations.

* P < 0.05 using Student's paired t-test to compare Turner syndrome at baseline and follow-up.

** P < 0.05 using Student's independent t-test to compare Turner syndrome at baseline to controls.

*** P > 0.05 using Student's independent t-test to compare Turner syndrome at baseline to controls

Aortic valve morphology could not be determined in one patient; BAV was present in 22 patients (28%) and tricuspid aortic valve (TAV) was seen in 57 patients (71%). The right and left coronary cusps were fused in 18 (82%) of patients with BAV, the right and non-coronary cusps were fused in 4 patients (18%), and none had fusion of the left and non-coronary cusps. In TS, the aortic valve was regurgitant in 17 patients (21% [15 were mild and 2 were moderate, while none were severe]) and stenotic in 9 patients (11% [7 were mild and 2 were moderate, while none were severe]). Aortic stenosis and regurgitation co-existed in 5 TS patients (6%), who all had BAV. Aortic valve regurgitation and stenosis (of any degree) were more likely to be present in patients with BAV than TAV (for aortic stenosis: 8 patients (36%) with BAV and 1 (2%) with TAV [p = 0.001], and for aortic regurgitation: 9 patients (41%) with BAV and 8 patients (14%) with TAV [p = 0.04]). Predefined arch anomalies are given in Table 1. All controls had normal aortic valve morphology and two (3%) had mild aortic valve regurgitation, while none had aortic stenosis.

Baseline aortic measurements were comparable in the 80 patients with TS, who participated in the follow-up, when compared to those who did not complete the follow-up.

### Aortic dimensions and growth

Absolute aortic diameters in TS and controls were comparable at all regions except the distal transverse aortic arch and the aortic isthmus, where the aorta was smaller in TS (p < 0.05). In contrast to this, BSA-indexed aortic diameters were enlarged at all positions in TS (p < 0.05). Only at the sinotubular junction was the prevalence of absolute aortic dilation higher in TS (10% in TS versus 2% in controls, p = 0.04), while the prevalence of BSA-indexed dilation was much more common (Table 2). BAV, aortic coarctation, elongated transverse aortic arch, blood pressure elevation (systolic and diastolic), age and karyotype 45X were associated with larger baseline aortic diameters in TS [6,14].

**Table 2:** Aortic dilation and aortic growth in Turner syndrome

	**Aortic dilation **^**a**^		Aortic diameter and growth			
	
	Turner syndrome	Controls	Turner syndrome			
			
			Baseline	Change	**Change rate **^**b**^	
	
	n = 80	n = 67	n = 80	n = 80	n = 80	n = 80
	
	%	%	mm	mm	mm/year	**mm/year/m**^**2**^
**Aortic sinus**	18.9*	1.5	29.2 ± 3.9	1.0 ± 1.9**	0.38 ± 0.7	0.26 ± 0.5
**Sinotubular junction**	30.3*	2.9	25.3 ± 4.3	0.4 ± 1.3**	0.11 ± 0.5	0.07 ± 0.3
**Mid-ascending aorta**	36.7*	1.5	27.5 ± 5.0	0.6 ± 1.4**	0.24 ± 0.6	0.16 ± 0.4
**Distal ascending aorta**	33.3*	2.9	25.3 ± 3.6	-0.1 ± 1.0	-0.01 ± 0.4	-0.01 ± 0.3
**Proximal transverse aortic arch**	24.1*	3.6	23.4 ± 3.6	0.1 ± 0.9	-0.01 ± 0.4	-0.01 ± 0.2
**Distal transverse aortic arch**	10.3	3.0	20.5 ± 2.7	0.1 ± 0.8	0.01 ± 0.4	0.01 ± 0.3
**Aortic isthmus**	14.1*	3.0	19.3 ± 2.3	0.1 ± 0.8	0.05 ± 0.4	0.03 ± 0.3
**Proximal descending aorta**	34.2*	1.5	19.5 ± 2.8	-0.1 ± 0.8	-0.01 ± 0.3	-0.01 ± 0.2
**Distal descending aorta**	30.7*	1.5	18.2 ± 2.2	-0.1 ± 0.6	-0.03 ± 0.3	-0.02 ± 0.2

Continuous variables are expressed as means ± standard deviations.

^a ^Aortic dilation is defined by the mean + 1.96 standard deviations in the controls, and calculated from body surface area indexed diameters.

^b ^Aortic growth rates are calculated as the individual change in diameter from baseline to follow-up weighted individually for the duration of the follow-up.

* P < 0.05 using chi-square or Fisher's exact 2-sided test to compare Turner syndrome to controls.

** P < 0.05 using Student's paired t-test to compare baseline aortic diameter to follow-up in Turner syndrome.

At follow-up increases in aortic diameter were limited to the aortic sinuses, the sinotubular junction and the mid-ascending aorta (Table 2). The presence of BAV associated with more pronounced aortic growth (absolute; follow-up time-weighted absolute; and indexed for both follow-up time and BSA) at sinus level only (BAV versus TAV: 1.64 ± 2.19 versus 0.69 ± 1.71 mm [p = 0.04]; 0.64 ± 0.84 versus 0.26 ± 0.62 mm/year [p = 0.04]; and 0.44 ± 0.57 versus 0.18 ± 0.61 mm/year/m^2 ^[p = 0.03]). The type of BAV cusp morphology did not associate with aortic growth. Age was comparable in patients with TAV and BAV (p = 0.9). Increments in aortic sinus diameter were not exclusive to patients with BAV, as they were also seen in TAV (baseline versus follow-up diameter in TAV: 28.5 ± 3.5 versus 29.2 ± 3.9 mm [p < 0.001]). No other aortic measurement position was found to have similar associations with BAV.

The principal determinants of the aortic growth rate were further evaluated by multiple linear regression models, using aortic growth rate as the dependent variable. The independent variables were chosen from baseline correlations with aortic diameter [6,14]. Models incorporating these variables were only significant at the level of the aortic sinus. One model (R = 0.26, p = 0.03) had aortic growth rate at sinus level as the dependent variable, where aortic valve morphology was the only explanatory variable, while age, 24-hour systolic blood pressure, BSA and antihypertensive treatment did not contribute.

Baseline aortic diameters, aortic dilation, aortic valve function and aortic coarctation were not related to aortic growth at any position (neither with or without indexing for follow-up time). The presence of an elongated transverse aortic arch was associated with lower growth rates (absolute and BSA-indexed) in the distal transverse aortic arch and proximal ascending aorta (p < 0.05). Karyotype 45X conferred less rapid growth rates in the distal aortic arch (p = 0.002) and in the proximal descending aorta (p = 0.006). None of the following variables were found to relate with aortic growth (absolute or BSA-indexed): age, estrogen replacement therapy, previous growth hormone treatment, baseline blood pressure, changes in blood pressures during follow-up or antihypertensive treatment.

In 31 (39%) of the TS patients the increase in aortic diameter exceeded the interobserver limits of agreement in one or more positions (but never in the proximal arch or the aortic isthmus). These mainly affected the ascending aorta (29/31 [94%]), involving the aortic sinus (24/31 [77%]), the sinotubular junction (6/31 [19%]) and the mid-ascending aorta (12/31 [39%]). Aortic valve morphology, aortic arch abnormalities, aortic dilation (absolute or BSA indexed) or antihypertensive treatment were not more common in patients with such apparently fast growing aortas. However, this subset did have marginally higher 24-hour heart rates (p = 0.08), whereas blood and pulse pressures (or changes in the 24-hour ambulatory blood pressure parameters during follow-up) were not different.

Nineteen patients (24%) had annual growth rates that exceeded two standard deviations above the mean change for the whole TS group; no risk marker was more prevalent in this group.

### Blood pressure and Heart rate

Baseline blood pressures were higher in TS than in controls even though 30% of the patients (24/80) took antihypertensive medication at baseline (Table 1). At follow up, the number of patients on antihypertensive medication had increased to 46% (37/80) with a concomitant blood pressure and heart rate reduction (Table 1). All antihypertensive medication was prescribed for elevated blood pressures rather than for aortic dilatation and dissection prophylaxis.

### Reproducibility of CMR

The intra- and interobserver measurement variability was low (Table 3). There was no systematic variation at any aortic region of interest to suggest bias, or with increasing or decreasing aortic diameter.

**Table 3:** Measurement variability of multiplanar aortic CMR measurements

	Intraobserver		Interobserver	
	
	**Mean difference **^**a**^	**Limits of agreement **^**b**^	**Mean difference **^**a**^	**Limits of agreement **^**b**^
	
	mm	mm	mm	mm
**Aortic sinus**	-0.04	-1.9 - 1.8	0.1	-2.1 - 2.3
**Sinotubular junction**	0.02	-1.8 - 1.9	-0.3	-2.3 - 1.8
**Mid-ascending aorta**	-0.1	-1.9 - 1.8	-0.1	-1.9 - 1.4
**Distal ascending aorta**	-0.1	-1.9 - 2.1	0.1	-1.6 - 1.7
**Proximal transverse aortic arch**	0.2	-1.6 - 2.0	-0.2	-1.4 - 1.9
**Distal transverse aortic arch**	0.01	-1.7 - 1.7	-0.01	-1.6 - 1.4
**Aortic isthmus**	0.1	-1.6 - 1.4	-0.1	-1.4 - 1.9
**Proximal descending aorta**	0.08	-1.5 - 1.4	0.08	-1.1 - 1.9
**Distal descending aorta**	-0.06	-1.6 - 1.7	0.1	-1.2 - 1.5

Linear Bland-Altman analysis of inter- and intraobserver variability in the setting of repeat, maximum aortic diameter measurements with CMR using multiplanar reformatting of 3D non-contrast, free-breathing aortograms.

^a ^Mean difference between the first and second measurement by the same observer, or between different observers.

^b ^The limits of agreement are the mean difference ± 1.96 standard deviations.

## Discussion

This prospective CMR study documents the progressive nature of aortic dilation in TS. In keeping with a propensity towards ascending aortic dilation and dissection in TS [4,5], the process of dilation was found to affect the aortic sinus, the sinotubular junction and the mid-ascending aorta. Furthermore, the present study provides novel evidence to the adverse impact of BAV morphology on aortic growth rates in TS, as only previously indicated by cross-sectional studies [6,9,12].

The only preceding, longitudinal evaluation of aortic dimensions in TS used TTE and failed to identify predictors of aortic growth [11]. However, limited acoustic windows in coexistence with a complex aortopathy in the entire thoracic aorta render TTE less beneficial in TS [12,13]. In contrast to TTE, the present CMR sequence facilitated a comprehensive assessment of the thoracic aorta without limitations of acoustic windows and with high-reproducibility post-processing of 3D data. The current CMR methodology thus provided optimised aortic assessment in TS, which should always include a complete view of the thoracic aorta even though we did not detect aortic growth beyond the mid-ascending aorta on whole group analysis. This need for evaluation of the entire thoracic aorta beyond the ascending aorta is justified in the present study not only by highly abnormal descending aortic diameters and very frequent pathology in the transverse aortic arch but also by the presence of increasing descending aortic diameters in some, individual TS patients in our cohort. Additionally, this need for a comprehensive delineation of aortic morphology is supported by the previously demonstrated occurrence of aortic dissections in the descending aorta in TS [4]. Consequently, TTE should in our opinion be restricted to follow-up in patients where aortic morphology is proven to be normal in the context of adequate acoustic windows. Our stance corresponds well with recent guidelines for diagnosis and monitoring of thoracic aortic disease, where TTE was deemed problematic for serial assessment of aortic calibre when pathology extends beyond the sinotubular junction [16]. Collectively, non-contrast and non-radiation investigations should be the gold standard for serial aortic assessment in TS, where imaging must provide sufficient aortic overview that is free from acoustic limitations and ideally provide reproducible 3D data. As a consequence of this, our method and findings are applicable beyond the research setting because 3D data was acquired without the use of radiation or contrast and with a short total scan time (on average 10-15 minutes). Furthermore, the present recruitment method ensured inclusion of a representative spectrum of TS phenotypes covering a wide age range, which makes our findings on aortic growth rates applicable as a CMR reference to the general adult TS population. Supporting this degree of external validity, the prevalence of congenital cardiac abnormalities was comparable to previous studies [20].

The presence of BAV was associated with more rapid growth in the aortic sinus than seen in patients with TAV. However, even though abnormal valve morphology was of prognostic importance, ascending aortic diameter also increased in patients with normal valve morphology over 2.4 years. Additionally, and irrespective of aortic valve morphology, the observed aortic growth rates in TS exceeded annual growth rates of 0.07 mm as previously demonstrated in healthy females using 2D CMR [21], and they were at least comparable to those demonstrated in patients with BAV morphology found to be 0.19 mm/year using a spectrum of imaging modalities [22]. Therefore, patients with TS appear to suffer from progressive aortopathy regardless of valve morphology in this relatively short-term follow-up study. An extended follow-up period could have revealed further associations between aortic growth and other risk markers of dissection beyond aortic valve morphology, which might include hypertension, karyotype 45,X or aortic coarctation. This would be in line with previous indications of a multifactorial aetiology of aortic disease in TS, where aortic dissections occur even in the absence of the presently acknowledged markers of aortic dissection [4]. Consequently, risk stratification for aortic dissection in TS should not solely be based on cross-sectional aortic diameter and the presence of fixed risk factors (BAV, hypertension, aortic coarctation and karyotype); it should also include assessment of changes in aortic diameter in TS to provide a more nuanced prognostication. This inclusion of longitudinally assessed aortic diameter in risk stratification for aortic dissection was also acknowledged by the recent consensus guidelines on thoracic aortic disease [16].

The limits for surgical prophylaxis for aortic dissection remain to be defined from larger prospective and hard-endpoint studies in order to provide firm evidence-based guidance for risk reduction in the aortopathy associated with TS. The issue of initiation and choice of medical prophylaxis through antihypertensive treatment also remains unresolved in TS [3]. In the present study, blood pressure levels were not associated with progressive aortic dilatation, even though aortic diameter did associate with blood pressure at baseline [6]. This absence of association between blood pressure (or changes herein during follow-up) and aortic growth rates could be caused by the fact that participation in a study with 24-hour blood pressure monitoring will diagnose new cases with hypertension and identify insufficiently treated hypertensive patients. Since the patients' physicians were recommended to commence or intensify antihypertensive treatment according to the blood pressure readings, a larger fraction of patients were therefore on antihypertensive medicines at follow-up with a range of pharmacological interventions instituted. This is highly likely to have influenced the association between blood pressure and the process of aortic dilation. Thus, despite the absence of association between aortic growth and blood pressure, we believe that it is prudent to initiate antihypertensive treatment with a low threshold in TS, and conclusions on efficacy of specific antihypertensive strategies can only be drawn from randomised controlled trials.

## Conclusions

An accelerated ascending aortopathy was present in adult TS patients with growth rates of 0.1 - 0.4 mm/year during 2.4 years of prospective CMR follow-up. The presence of a BAV was associated with higher aortic growth rates, whereas other markers of aortic dilation and dissection did not correlate with aortic growth rates in non-selected TS.

## Competing interests

The authors declare that they have no competing interests.

## Authors' contributions

CHG and JSC conceived the study, with contributions to the design, coordination and conduction from KHM, BHE, KS, NHA, KES, AH, EL and EMP. CMR was performed and handled by KHM, EMP and EL. TTE was performed by KHM, NHA and KES. KHM, CG and NHA performed the statistical analyses and drafted the manuscript with substantial contributions from all other authors. All authors read and approved the final manuscript.

## References

[B1] Schoemaker MJ Swerdlow AJ Higgins CD Wright AF Jacobs PA Mortality in women with turner syndrome in Great Britain: a national cohort study J Clin Endocrinol Metab 2008 93 4735 4742 10.1210/jc.2008-1049 18812477

[B2] Stochholm K Juul S Juel K Naeraa RW Gravholt CH Prevalence, incidence, diagnostic delay, and mortality in Turner syndrome J Clin Endocrinol Metab 2006 91 3897 3902 10.1210/jc.2006-0558 16849410

[B3] Bondy CA Aortic dissection in Turner syndrome Curr Opin Cardiol 2008 23 519 526 10.1097/HCO.0b013e3283129b89 18839441 PMC2692924

[B4] Carlson M Silberbach M Dissection of the aorta in Turner syndrome: two cases and review of 85 cases in the literature J Med Genet 2007 44 745 749 10.1136/jmg.2007.052019 17873120 PMC2652808

[B5] Gravholt CH Landin-Wilhelmsen K Stochholm K Hjerrild BE Ledet T Djurhuus CB Sylvén L Baandrup U Kristensen BØ Christiansen JS Clinical and epidemiological description of aortic dissection in Turner's syndrome Cardiol Young 2006 16 430 436 10.1017/S1047951106000928 16984695

[B6] Hjerrild BE Mortensen KH Sorensen KE Pedersen EM Andersen NH Lundorf E Hansen KW Hørlyck A Hager A Christiansen JS Gravholt CH Thoracic aortopathy in Turner syndrome and the influence of bicuspid aortic valves and blood pressure: a CMR study J Cardiovasc Magn Reson 2010 12 12 10.1186/1532-429X-12-12 20222980 PMC2847561

[B7] Matura LA Ho VB Rosing DR Bondy CA Aortic dilatation and dissection in Turner syndrome Circulation 2007 116 1663 1670 10.1161/CIRCULATIONAHA.106.685487 17875973

[B8] Lopez L Arheart KL Colan SD Stein NS Lopez-Mitnik G Lin AE Reller MD Ventura R Silberbach M Turner syndrome is an independent risk factor for aortic dilation in the young Pediatrics 2008 121 e1622 e1627 10.1542/peds.2007-2807 18504294

[B9] Cleemann L Mortensen KH Holm K Smedegaard H Skouby SO Wieslander SB Leffers AM Leth-Espensen P Pedersen EM Gravholt CH Aortic Dimensions in Girls and Young Women with Turner Syndrome: A Magnetic Resonance Imaging Study Pediatr Cardiol 2010 10.1007/s00246-009-9626-8 20063160

[B10] Elsheikh M Casadei B Conway GS Wass JA Hypertension is a major risk factor for aortic root dilatation in women with Turner's syndrome Clin Endocrinol (Oxf) 2001 54 69 73 10.1046/j.1365-2265.2001.01154.x 11167928

[B11] Lanzarini L Larizza D Prete G Calcaterra V Klersy C Prospective evaluation of aortic dimensions in Turner syndrome: a 2-dimensional echocardiographic study J Am Soc Echocardiogr 2007 20 307 313 10.1016/j.echo.2006.08.028 17336759

[B12] Sachdev V Matura LA Sidenko S Ho VB Arai AE Rosing DR Bondy CA Aortic valve disease in Turner syndrome J Am Coll Cardiol 2008 51 1904 1909 10.1016/j.jacc.2008.02.035 18466808

[B13] Ostberg JE Brookes JA McCarthy C Halcox J Conway GS A comparison of echocardiography and magnetic resonance imaging in cardiovascular screening of adults with Turner syndrome J Clin Endocrinol Metab 2004 89 5966 5971 10.1210/jc.2004-1090 15579745

[B14] Mortensen KH Hjerrild BE Andersen NH Sorensen KE Horlyck A Pedersen EM Lundorf E Christiansen JS Gravholt CH Abnormalities of the major intrathoracic arteries in Turner syndrome as revealed by magnetic resonance imaging Cardiol Young 2010 1 10 10.1017/S1047951110000041 20307329

[B15] Sorensen TS Korperich H Greil GF Eichhorn J Barth P Meyer H Pedersen EM Beerbaum P Operator-independent isotropic three-dimensional magnetic resonance imaging for morphology in congenital heart disease: a validation study Circulation 2004 110 163 169 10.1161/01.CIR.0000134282.35183.AD 15210590

[B16] Hiratzka LF Bakris GL Beckman JA Bersin RM Carr VF Casey DE Jr Eagle KA Hermann LK Isselbacher EM Kazerooni EA Kouchoukos NT Lytle BW Milewicz DM Reich DL Sen S Shinn JA Svensson LG Williams DM American College of Cardiology Foundation/American Heart Association Task Force on Practice Guidelines; American Association for Thoracic Surgery; American College of Radiology; American Stroke Association; Society of Cardiovascular Anesthesiologists; Society for Cardiovascular Angiography and Interventions; Society of Interventional Radiology; Society of Thoracic Surgeons; Society for Vascular Medicine 2010 ACCF/AHA/AATS/ACR/ASA/SCA/SCAI/SIR/STS/SVM guidelines for the diagnosis and management of patients with Thoracic Aortic Disease: a report of the American College of Cardiology Foundation/American Heart Association Task Force on Practice Guidelines, American Association for Thoracic Surgery, American College of Radiology, American Stroke Association, Society of Cardiovascular Anesthesiologists, Society for Cardiovascular Angiography and Interventions, Society of Interventional Radiology, Society of Thoracic Surgeons, and Society for Vascular Medicine Circulation 2010 121 e266 e369 20233780 10.1161/CIR.0b013e3181d4739e

[B17] Schaefer BM Lewin MB Stout KK Gill E Prueitt A Byers PH Otto CM The bicuspid aortic valve: an integrated phenotypic classification of leaflet morphology and aortic root shape Heart 2008 94 1634 1638 10.1136/hrt.2007.132092 18308868

[B18] American College of Cardiology/American Heart Association Task Force on Practice Guidelines; Society of Cardiovascular Anesthesiologists; Society for Cardiovascular Angiography and Interventions; Society of Thoracic Surgeons Bonow RO Carabello BA Kanu C de Leon AC Jr Faxon DP Freed MD Gaasch WH Lytle BW Nishimura RA O'Gara PT O'Rourke RA Otto CM Shah PM Shanewise JS Smith SC Jr Jacobs AK Adams CD Anderson JL Antman EM Faxon DP Fuster V Halperin JL Hiratzka LF Hunt SA Lytle BW Nishimura R Page RL Riegel B ACC/AHA 2006 guidelines for the management of patients with valvular heart disease: a report of the American College of Cardiology/American Heart Association Task Force on Practice Guidelines (writing committee to revise the 1998 Guidelines for the Management of Patients With Valvular Heart Disease): developed in collaboration with the Society of Cardiovascular Anesthesiologists: endorsed by the Society for Cardiovascular Angiography and Interventions and the Society of Thoracic Surgeons Circulation 2006 114 e84 231 10.1161/CIRCULATIONAHA.106.176857 16880336

[B19] Bland JM Altman DG Measuring agreement in method comparison studies Stat Methods Med Res 1999 8 135 160 10.1191/096228099673819272 10501650

[B20] Ho VB Bakalov VK Cooley M Van PL Hood MN Burklow TR Bondy CA Major vascular anomalies in Turner syndrome: prevalence and magnetic resonance angiographic features Circulation 2004 110 1694 1700 10.1161/01.CIR.0000142290.35842.B0 15353492

[B21] Burman ED Keegan J Kilner PJ Aortic root measurement by cardiovascular magnetic resonance: specification of planes and lines of measurement and corresponding normal values Circ Cardiovasc Imaging 2008 1 104 113 10.1161/CIRCIMAGING.108.768911 19808527

[B22] Davies RR Kaple RK Mandapati D Gallo A Botta DM Jr Elefteriades JA Coady MA Natural history of ascending aortic aneurysms in the setting of an unreplaced bicuspid aortic valve Ann Thorac Surg 2007 83 1338 1344 10.1016/j.athoracsur.2006.10.074 17383337

[B23] Hjerrild BE Mortensen KH Gravholt CH Turner syndrome and clinical treatment Br Med Bull 2008 86 77 93 10.1093/bmb/ldn015 18400842

